# Sonography to Rule Out Tuberculosis in Sub-Saharan Africa: A Prospective Observational Study

**DOI:** 10.1093/ofid/ofz154

**Published:** 2019-04-25

**Authors:** Robert Ndege, Maja Weisser, Luigia Elzi, Flavia Diggelmann, Farida Bani, Winfrid Gingo, George Sikalengo, Herry Mapesi, Elisante Mchomvu, Lujeko Kamwela, Dorcas Mnzava, Manuel Battegay, Klaus Reither, Daniel H Paris, Martin Rohacek

**Affiliations:** 1 Ifakara Health Institute, United Republic of Tanzania; 2 St. Francis Referral Hospital, Ifakara, United Republic of Tanzania; 3 Division of Infectious Diseases, University Hospital Basel, Switzerland; 4 Department of Medicine, Swiss Tropical and Public Health Institute, Basel, Switzerland; 5 Faculty of Medicine, University of Basel, Switzerland; 6 Regional Hospital of Bellinzona e Valli, Switzerland

**Keywords:** FASH, sonography, sub-Saharan Africa, tuberculosis

## Abstract

**Background:**

Patients with suspected tuberculosis are often overtreated with antituberculosis drugs. We evaluated the diagnostic value of the focused assessment with sonography for HIV-associated tuberculosis (FASH) in rural Tanzania.

**Methods:**

In a prospective cohort study, the frequency of FASH signs was compared between patients with confirmed tuberculosis and those without tuberculosis. Clinical and laboratory examination, chest x-ray, Xpert MTB/RIF assay, and culture from sputum, sterile body fluids, lymph node aspirates, and Xpert MTB/RIF urine assay was done.

**Results:**

Of 191 analyzed patients with a 6-month follow-up, 52.4% tested positive for human immunodeficiency virus, 21.5% had clinically suspected pulmonary tuberculosis, 3.7% had extrapulmonary tuberculosis, and 74.9% had extrapulmonary and pulmonary tuberculosis. Tuberculosis was microbiologically confirmed in 57.6%, probable in 13.1%, and excluded in 29.3%. Ten of eleven patients with splenic or hepatic hypoechogenic lesions had confirmed tuberculosis. In a univariate model, abdominal lymphadenopathy was significantly associated with confirmed tuberculosis. Pleural- and pericardial effusion, ascites, and thickened ileum wall lacked significant association. In a multiple regression model, abnormal chest x-ray (odds ratio [OR] = 6.19; 95% confidence interval [CI], 1.96–19.6; *P* < .002), ≥1 FASH-sign (OR = 3.33; 95% CI, 1.21–9.12; *P* = .019), and body temperature (OR = 2.48; 95% CI, 1.52–5.03; *P* = .001 per °C increase) remained associated with tuberculosis. A combination of ≥1 FASH sign, abnormal chest x-ray, and temperature ≥37.5°C had 99.1% sensitivity (95% CI, 94.9–99.9), 35.2% specificity (95% CI, 22.7–49.4), and a positive and negative predictive value of 75.2% (95% CI, 71.3–78.7) and 95.0% (95% CI, 72.3–99.3).

**Conclusions:**

The absence of FASH signs combined with a normal chest x-ray and body temperature <37.5°C might exclude tuberculosis.

Tuberculosis remains one of the world’s deadliest communicable diseases. In 2017, an estimated 10 million people fell ill with tuberculosis worldwide, and 1.6 million died of the disease [1]. In resource-limited settings with high tuberculosis prevalence, up to half of patients with suspected disease are treated with antituberculosis drugs without microbiological confirmation, and 30%–60% of these empirically treated patients do not have active tuberculosis [2, 3]. Among reasons for the decision to treat without a positive test result are limited availability and reduced sensitivity of microbiological tests, particularly in patients coinfected with human immunodeficiency virus (HIV) or with suspected extrapulmonary tuberculosis [4–8]. The overtreatment of patients without tuberculosis is typically followed by a delay or lack of diagnosis of the real underlying disease, causing unnecessary drug exposure, side effects, and costs.

Focused Assessment with Sonography for HIV-associated Tuberculosis (FASH) was developed as a method to detect signs of extrapulmonary tuberculosis, which can be learned and performed by personnel with little or no previous experience in ultrasonography [9]. The FASH consists of a rapid sonographic evaluation of the abdomen, pleural space, and heart, to identify pleural and pericardial effusion, abdominal lymphadenopathy, hypoechogenic lesions in the spleen and the liver, ascites, and thickening of the bowel wall [10]. Some of these signs have been reported to occur in the majority of HIV-positive patients with suspected extrapulmonary tuberculosis, but tuberculosis was microbiologically confirmed in only a minority of those patients [11–14].

The diagnostic value of FASH in patients with tuberculosis has not been evaluated to date [15]. To address this need, we performed a prospective observational cohort study of patients with suspected tuberculosis and 6-month follow-up in a rural region in Tanzania. In particular, we aimed (1) to compare the frequency of sonographic signs of extrapulmonary tuberculosis in patients with confirmed tuberculosis to patients with no tuberculosis and (2) to determine the sensitivity, specificity, and predictive values of FASH.

## METHODS

### Study Site

This prospective observational study was performed at the St. Francis Referral Hospital in Ifakara, Tanzania. The hospital serves a rural population of approximately 1 million people living in the Kilombero, Ulang, and Malinyi districts as a referral center. The hospital has 360 beds, runs an emergency department, and includes the Chronic Diseases Clinic Ifakara, which specializes in HIV and tuberculosis management [16, 17].

### Study Population

Human immunodeficiency virus-positive and -negative adults aged ≥18 years presenting to any department of the St. Francis Referral Hospital or to the Chronic Diseases Clinic of Ifakara and fulfilling the following criteria were eligible: (1) suspected pulmonary tuberculosis, defined as presence of fever of any duration and/or night sweats during 3 weeks within the last 4 weeks, and/or weight loss, together with cough of any duration and/or hemoptysis or infiltrate on chest x-ray; (2) suspected extrapulmonary tuberculosis, defined as presence of ≥1 of the following signs or symptoms: fever of any duration, night sweats during 3 weeks within the last 4 weeks, weight loss, lymphadenopathy, abdominal pain, or ascites, or neurological symptoms, presence of severe anemia (hemoglobin <8 g/dL) in an HIV-infected patient under antiretroviral treatment [18], with no cough, infiltrate in chest x-ray, or other obvious clinical explanation for those signs and symptoms listed above; (3) suspected combination of pulmonary and extrapulmonary tuberculosis, defined as presence of fever of any duration and/or night sweats during 3 weeks within the last 4 weeks, and/or weight loss, together with pulmonary and extrapulmonary signs and symptoms. Pregnant women, patients who refused to participate, and patients under pre-existing treatment with antituberculosis drugs were excluded.

### Study Procedures

Patients were consecutively recruited from routine care during 1 year starting from July 2016. All included patients were interviewed and examined according to a standardized, predefined questionnaire. Axillary body temperature was measured with a digital thermometer. Chest x-ray with posterior-anterior projection was performed on the day of enrollment together with an HIV test (SD Bioline HIV 1/2 3.0; Abbott), which was confirmed by a Uni-Gold HIV Rapid Test (Trinity Biotech), if positive. Blood tests for hemoglobin, creatinine, and alanine aminotransferase were performed using Reflotron (Roche, Basel, Switzerland). All chest x-rays were reviewed and analyzed by R.N., M.W., and M.R., the last 2 of whom are board-certified physicians. Unclear findings were discussed and interpreted by this team.

At enrollment, early morning urine and sputum samples were collected from all patients. If spontaneous sputum was not available, sputum was induced by inhalation of nebulized 3% saline solution. To ensure consistent sputum quality, a video (https://www.youtube.com/watch?v=2 sd2d2_pNBA) on how to deliver sputum was shown to all patients. In case of pleural or pericardial effusion, ascites, lymphadenopathy, or meningitis, ultrasound-guided puncture and fluid aspiration was performed.

All sputum and aspirated fluid samples were analyzed by Xpert MTB/RIF assay (Cepheid, Sunnyvale, CA). In addition, sputum was processed by adding cetylpiridium chloride and *N*-acetyl-l-cysteine-sodium hydroxide and inoculated on Löwenstein-Jensen medium [19]. Sterile fluids (pleural, pericardial, and cerebrospinal fluids, ascites, and lymph node aspirates) were inoculated into liquid culture using bacteria growth indicator tube (MGIT) with a BACTEC 960 Instrument (BD Microbiology Systems, Sparks, MD). Urine was examined with Xpert MTB/RIF assay after centrifugation. Adenosine deaminase (Diazyme, Poway, CA) was measured in pleural, ascitic, and pericardial fluids.

All patients received a sonographic examination according to the FASH protocol (see Supplementary Table S1), which was performed by R.N. and F.D. The examination was supervised by M.R., either in person or by reviewing recorded images and video clips. R.N. and M.R. are board-certified sonographers. All examinations were performed with a Mindray M7 ultrasound machine (Mindray, Shenzhen, China). A convex array probe C5-2s was used, except for sonography of the ileum, where a linear array 7L-4s probe was used. All sonographic examinations were done prior to sample taking, and microbiological results were not available to the sonographers. Clinical information could not be masked to the sonographers, but it was masked to the laboratory personnel performing microbiological tests.

All data were entered into standardized electronic questionnaires (EpiData). During follow-up, decisions on whether to administer antituberculosis treatment were considered by the patients’ treating physicians, who were not blinded to the results of the sonographic examination. Patients were scheduled for follow-up visits 2 and 6 months after enrollment. If patients missed their appointment, they were traced by repeated phone calls or physically by visiting their homes by motorcycle. During follow-up visits, clinical evaluation and FASH were done at 2 and 6 months.

### Definitions

#### FASH Signs

 Original FASH signs consisted of pleural or pericardial effusion, ascites, abdominal lymph nodes >1.5 cm, hyperechogenic lesions in the liver or spleen, ileum wall thickening >4 mm, or destructed ileum wall architecture. In addition, we systematically evaluated the presence of splenomegaly, hepatomegaly, and pleural or pericardial fibrin strands in presence of effusion.

Confirmed tuberculosis was defined as ≥1 positive microbiological result from any site confirmed by Xpert MTB/RIF assay and/or bacteriologic culture (growth of *Mycobacterium tuberculosis*) in sputum, pleural fluid, ascites, cerebrospinal fluid, urine, or lymph node aspirate. In addition, the identification of acid-fast bacilli in sputum by another health center or adenosine deaminase (ADA) ≥40 U/mL in pleural fluid [20], ≥35 U/mL in pericardial fluid [4], and ≥30 U/mL in ascitic fluid [21] were accepted as microbiological confirmation.

Probable tuberculosis was defined as negative microbiological tests in a patient in whom antituberculosis therapy (prescribed based on clinical suspicion or on chest x-ray) in the absence of an alternative diagnosis led to a resolution of clinical signs and symptoms, radiographic and sonographic signs, and to an increase in body weight documented 2 months after start of antituberculosis treatment.

No tuberculosis was defined as consistently negative microbiological tests in a patient in whom signs and symptoms, radiographic, and sonographic signs subsided during follow-up and body weight increased without antituberculosis treatment, or if a patient with negative microbiological results had no resolution of clinical, radiographic, or sonographic signs during follow-up despite empirical antituberculosis treatment.

Abnormal chest x-ray was defined as the presence of upper lobe or any other infiltrate, cavernous lesion, miliary infiltrates, or pleural effusion on chest radiogram in posterior to anterior projection.

### Statistical Analysis

Basic demographic characteristics, clinical and laboratory parameters, chest x-ray, and FASH signs were compared according to diagnosis of (1) confirmed tuberculosis versus no tuberculosis and (2) confirmed or probable tuberculosis versus no tuberculosis. We chose confirmed tuberculosis as the primary outcome, because it reflects the microbiological reference standard. As a secondary outcome, we chose the composite reference standard of confirmed or probable tuberculosis as a clinically relevant outcome [22]. We used χ^2^ test or Fisher’s exact test for categorical variables and the Mann-Whitney *U* test for continuous variables. Logistic regression was used to estimate the prediction of confirmed and confirmed or probable tuberculosis according to different clinical and radiological criteria including FASH. A backward stepwise multivariable logistic regression analysis on the selected variables to form the prediction model (entry criteria = *P* < .05; removal criteria = *P* ≥ .10) was used. We retained those variables that are known to be associated with tuberculosis (younger age, male sex, HIV infection, lower body mass index). Likelihood ratio tests were used to measure goodness of the fit of the regression models. Results are presented as crude and adjusted odds ratios (ORs) after adjusting for potential confounders as indicated. Finally, we checked the models for any interactions. Patients with missing baseline- or follow-up data were excluded from analysis. All analyses were performed using STATA software version 13 for Windows (Stata Corp., College Station, TX).

### Ethical Statement

The study was approved by the Swiss ethics committee Ethikkommission Nordwest und Zentralschweiz (Nr 2015/243) and the ethics committees of the Ifakara Health Institute (Institutional Review Board, IHI/IRB/No 02-2016) and the National Institute for Medical Research, Tanzania (Ref. NIMR/HQ/R.8a/Vol. IX/2244). All participants signed an informed consent form.

## RESULTS

**Figure 1.**
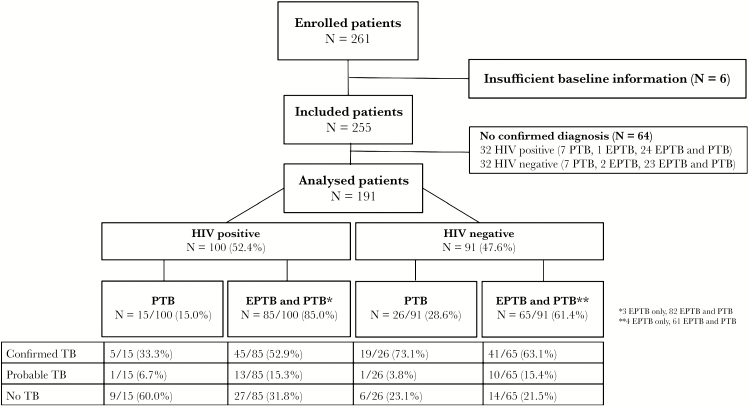
Enrollment of patients. Patients are presented according human immunodeficiency virus (HIV) status and final diagnosis reached. No confirmed diagnosis, no confirmed tuberculosis (TB) at inclusion, and lost to follow-up; confirmed TB, microbiologically confirmed TB; PTB, pulmonary tuberculosis; EPTB, extrapulmonary tuberculosis; EPTB and PTB, presence of EPTB and PTB in a patient.

From July 2016 through to June 2017, 261 patients were enrolled. Seventy patients were excluded: 64 with negative microbiological results at baseline and insufficient follow-up information to classify them as confirmed, probable, or no tuberculosis, and 6 with insufficient baseline information. Overall, 191 patients were included in the final analysis; of these, 100 (52.4%) were HIV-positive and 36 (18.8%) were hospitalized. A total of 41 (21.5%) patients were clinically suspected to have pulmonary tuberculosis only, 7 (3.7%) were suspected to have extrapulmonary tuberculosis only, and 143 (74.9%) were suspected to have combined extrapulmonary and pulmonary tuberculosis. After 6 months follow-up, 110 (57.6%) patients had a final diagnosis of confirmed tuberculosis, 25 (13.1%) had a diagnosis of probable tuberculosis, and 56 (29.3%) had no tuberculosis (Figure 1).

Table 1 shows baseline characteristics according to the final diagnosis. Compared to patients with no tuberculosis, patients with confirmed tuberculosis were younger, were more likely to be male, had a lower body mass index, reported weight loss more frequently, were less likely to be HIV-positive, and had distinct clinical presentations (higher temperature, lower oxygen saturation, more frequent lymphadenopathy, and abnormal chest x-ray). On sonography, patients with confirmed tuberculosis had significantly more signs of pleural effusion, pleural fibrin strands, abdominal lymphadenopathy, hepatomegaly, or ≥1 original FASH sign than patients with no tuberculosis.

**Table 1.** Patient’s Characteristics According to Diagnosis Confirmed TB Versus No TB

		Confirmed TB		No TB		
		N = 110		N = 56		
Characteristic		N	% or IQR	N	% or IQR	*P* Value
Median age, years		36.5	27.1–43.3	41.5	34.2–57.3	**.002**
Male sex		70	63.6	25	44.6	**.019**
Body mass index, kg/m^2^		18.3	16.7–20.6	21.2	19.1–23.8	**<.001**
HIV infection		50	45.5	36	64.3	**.022**
Symptoms	Fever	74	67.3	36	64.3	.700
	Cough	104	94.5	49	87.5	.110
	Hemoptysis	20	18.8	10	17.9	.959
	Dyspnea	55	50.0	30	53.6	.663
	Chest pain	67	60.9	40	71.4	.181
	Night sweats	74	67.3	29	51.8	.052
	Weight loss	86	78.9	33	62.3	**.024**
	Abdominal symptoms	44	40.0	40	71.4	**<.001**
	Neurological symptoms	29	26.4	15	26.8	.954
Clinical signs	Median temperature, °C	37.4	36.7–38.0	36.6	36.0–37.0	**<.001**
	Median SaO_2_	97	95–98	98	96–98	**.026**
	Pulmonary signs^a^	76	69.1	28	50.0	**.016**
	Cardiac signs^b^	8	7.3	6	10.7	.451
	Abdominal signs^c^	64	58.2	33	58.9	.926
	Lymphadenopathy^d^	70	63.4	24	42.9	**.011**
Chest x-ray	Upper lobe infiltrates	64	58.2	12	21.4	**<.001**
	Cavernous lesion	36	32.7	1	1.8	**<.001**
	Miliary infiltrates	3	2.2	1	1.8	.664
	Other infiltrates	65	59.0	21	37.5	.077
	Pleural effusion	23	20.9	6	10.7	.111
Clinical TB	PTB	24	21.8	15	26.8	.061
	EPTB	2	1.8	5	8.9	
	EPTB and PTB	84	76.4	36	64.3	
Original FASH signs	Pleural effusion	26	23.6	6	10.7	**.046**
	Pericardial effusion	20	18.8	5	8.9	.086
	Ascites	28	25.5	11	19.6	.404
	Abdominal LN	23	20.9	2	3.6	**.002**
	Hypoechogenic lesions in liver/spleen	10	9.1	0	-	-
	Ileum wall thickening	7	6.4	3	5.4	.604
	Ileum wall destruction	6	5.4	4	7.1	.410
Additional sonographic signs	Splenomegaly	29	26.3	16	29.1	.711
	Hepatomegaly	58	52.7	20	35.7	**.038**
	Pleural fibrin starnds	14	12.7	2	3.6	**.047**
	Pericardial fibrin strands	2	1.8	0	-	-
≥1 original FASH sign		61	55.5	16	28.6	**.001**
≥1 sonographic sign^e^		88	80.0	38	67.9	.084
Number of sonographic signs^e^	0	22	20.0	18	32.1	**.038**
	1	34	30.9	21	37.5	
	2	16	14.6	7	12.5	
	≥3	38	34.5	10	17.9	

*P* values in bold numbers indicate that the difference is statistically significant.

Abbreviations: abdominal LN, abdominal lymph nodes >1.5 cm; EPTB, extrapulmonary tuberculosis; FASH, focused assessment with sonography for HIV-associated tuberculosis; HIV, human immunodeficiency virus; IQR, interquartile range; LN, lymph nodes; PTB, pulmonary tuberculosis, SaO_2_, oxygen saturation; TB, tuberculosis.

^a^Pulmonary signs included crackles, wheezing, pleural friction in lung auscultation, or dullness in lung percussion.

^b^Cardiac signs were dilated jugular veins, lateralized apex beat, or heart murmur.

^c^Abdominal signs included organomegaly, ascites, and abnormal bowel sound.

^d^Lymphadenopathy was diagnosed if palpable enlarged axillary, cervical, or nuchal lymph nodes were present on physical examination.

^e^Presence of at least any of the original FASH sign and/or splenomegaly, hepatomegaly, or pleural- or pericardial fibrin strands.

Of the 11 (6%) patients with hypoechogenic lesions in the liver or spleen (median diameter 10.3 mm, range 4–32 mm), all but 1 were HIV-positive, and all had confirmed (n = 10) or probable (n = 1) tuberculosis. There was no difference in the occurrence of ascites between the 2 groups. Of the 110 patients with confirmed tuberculosis, only 7 (6.3%) had a body temperature below 37.5°C together with a normal chest x-ray, and 6 of these had a positive FASH. When patients with confirmed or probable tuberculosis were compared with patients without tuberculosis, results were similar (see Supplementary Table S2). Supplementary Table S3 shows the baseline characteristics of the 64 patients lost to follow-up.

### Association of Clinical, Radiographic, and Sonographic Signs With Confirmed Tuberculosis

Univariate and multivariate regression models of predictors of confirmed tuberculosis versus no tuberculosis are shown in Tables 2 and 3, respectively. In the univariate model, older age (OR = 0.67; 95% confidence interval [CI], 0.53–0.85; *P* < .001), female sex (OR = 0.46; 95% CI, 0.24–0.88; *P* = .020), body mass index (OR = 0.37; 95% CI, 0.23–0.59 ; *P* < .001, per 5 kg/m^2^ increase), HIV infection (OR = 0.46; 95% CI, 0.24–0.89; *P* = .023), and presence of abdominal symptoms (OR = 0.27; 95% CI, 0.13–0.53; *P* < .001) were negatively associated with confirmed tuberculosis. Higher body temperature (OR = 3.2; 95% CI, 2.00–5.12; *P* < .001) and abnormal chest x-ray (OR = 6.32; 95% CI, 2.83–14.2; *P* < .001) were positively associated with confirmed tuberculosis. From the FASH signs, abdominal lymphadenopathy (OR = 7.14; 95% CI, 1.62–31.5; *P* = .009) and ≥1 original FASH sign (OR = 3.11; 95% CI, 1.56–6.21; *P* = .001) was associated with confirmed tuberculosis. We found no significant association between pleural effusion, pericardial effusion, ascites, or thickened ileum wall and confirmed tuberculosis. In the multivariate regression model, the association of increased body temperature (OR = 2.48; 95% CI, 1.52–5.03; *P* = .001 per each °C increase), pathological chest x-ray (OR = 6.19; 95% CI, 1.96–19.6; *P* = .002), and ≥1 original FASH signs (OR = 3.33; 95% CI, 1.21–9.12; *P* = .019) remained significant with confirmed tuberculosis. Supplementary Tables S4 and S5 show the univariate and multivariate regression models of predictors of the composite outcome of confirmed or probable tuberculosis versus no tuberculosis. The results were similar except that pleural effusion in FASH (OR = 3.63; 95% CI, 1.44–9.14; *P* = .006) and pleural fibrin strands (OR = 5.84; 95% CI, 1.33–25.6; *P* = .019) were significantly associated with confirmed or probable tuberculosis in the univariate model. Analyzing only patients with signs of extrapulmonary tuberculosis (N = 150), no relevant difference was found (data not shown).

**Table 2.** Predictors of Confirmed Tuberculosis Versus No Tuberculosis (Univariate Logistic Regression)

Predictor		Odds Ratios	95% CI	*P* Value
Age, per 10 years older		0.67	0.53–0.85	**<.001**
Female versus male		0.46	0.24–0.88	**.020**
Body mass index, per 5 kg/m^2^ increase		0.37	0.23–0.59	**<.001**
HIV infection		0.46	0.24–0.89	**.023**
Fever		1.14	0.58–2.24	.700
Cough		2.47	0.79–7.76	.120
Hemoptysis		1.02	0.44–2.36	.959
Dyspnea		0.87	0.45–1.65	.663
Chest pain		0.62	0.31–1.25	.182
Night sweats		1.91	0.99–3.69	.053
Weight loss		2.26	1.10–4.66	**.026**
Abdominal symptoms		0.27	0.13–0.53	**<.001**
Neurological symptoms		0.98	0.47–2.03	.954
Temperature, per each °C increase		3.20	2.00–5.12	**<.001**
SaO_2_		0.87	0.75–1.02	.071
Pulmonary signs^a^		2.23	1.15–4.33	**.017**
Cardiac signs^b^		0.65	0.22–1.98	.453
Abdominal signs^c^		0.97	0.50–1.86	.926
Lymphadenopathy^d^		2.33	1.20–4.49	**.011**
Chest x-ray	Upper lobe infiltrates	5.09	2.41–10.8	**<.001**
	Cavernous lesion	26.9	3.57–202.3	**.001**
	Miliary infiltrates	1.54	0.15–15.3	.710
	Other infiltrates	2.37	1.22–4.64	**.011**
	Pleural effusion	2.16	0.82–5.68	.117
	Abnormal chest x-ray	6.32	2.83–14.2	**<.001**
Original FASH signs	Pleural effusion	3.58	0.99–6.70	.052
	Pericardial effusion	2.27	0.80–6.40	.122
	Ascites	1.39	0.63–3.07	.405
	Abdominal LN	7.14	1.62–31.5	**.009**
	Mesenterial LN	1.77	1.27–2.45	**.001**
Additional sonographic signs	Splenomegaly	0.87	0.41–1.79	.711
	Hepatomegaly	2.01	1.03–3.89	**.039**
	Pleural fibrin	3.94	0.86–17.9	.077
	Ileum wall thickening	1.20	0.29–4.83	.797
	Ileum wall destruction	0.50	0.20–2.77	.666
≥1 original FASH sign		3.11	1.56–6.21	**.001**
Number of any sonographic sign^e^	0	-	-	-
	1	1.32	0.58–3.02	.505
	2	1.87	0.63–5.53	.258
	≥3	3.11	1.22–7.91	**.017**

*P* values in bold numbers indicate that the association is statistically significant.

Abbreviations: CI, confidence interval; EPTB, extrapulmonary tuberculosis; FASH, focused assessment with sonography for HIV-associated tuberculosis; HIV, human immunodeficiency virus; LN, lymph nodes; PTB pulmonary tuberculosis; SaO_2_, oxygen saturation.

^a^Pulmonary signs included crackles, wheezing, pleural friction in lung auscultation, or dullness in lung percussion.

^b^Cardiac signs included dilated jugular veins, lateralised apex beat, or heart murmur.

^c^Abdominal signs were organomegaly, ascites, and abnormal bowel sounds.

^d^Lymphadenopathy included palpable enlarged axillary, cervical, or nuchal lymph nodes on physical examination.

^e^Presence of at least any of the original FASH criteria and/or splenomegaly, hepatomegaly, or pleural- or pericardial fibrin strands.

**Table 3.** Predictors of Confirmed Tuberculosis (n = 110 vs 56): Multivariate Logistic Regression

Predictor	Odds Ratios	95% CI	*P* Value
Age, per 10 years older	0.72	0.51–1.00	.054
Female versus male	0.75	0.38–2.67	.971
Body mass index, per 5 kg/m^2^ increase	0.87	0.74–1.03	.087
HIV infection	0.61	0.21–1.75	.361
Cough	2.63	0.50–13.7	.251
Night sweats	0.68	0.25–1.84	.444
Weight loss	0.77	0.22–2.74	.688
Temperature, per each °C increase	2.48	1.52–5.03	**.001**
Pulmonary signs^a^	1.10	0.39–3.05	.859
Abdominal signs^b^	0.71	0.26–1.90	.493
Lymphadenopathy^c^	1.68	0.58–4.83	.338
Abnormal chest x-ray^d^	6.19	1.96–19.6	**.002**
≥1 FASH sign	3.33	1.21–9.12	**.019**

Odds ratios were adjusted for all variables listed; ≥1 FASH sign, presence of at least 1 original FASH sign. *P* values in bold numbers indicate that the association is statistically significant.

Abbreviations: CI, confidence interval; FASH, focused assessment with sonography for HIV-associated tuberculosis; HIV, human immunodeficiency virus.

^a^Pulmonary signs included crackles, wheezing, pleural friction in lung auscultation, or dullness in lung percussion.

^b^Abdominal signs included organomegaly, ascites, and abnormal bowel sound.

^c^Lymphadenopathy was diagnosed if palpable enlarged axillary, cervical, or nuchal lymph nodes were present on physical examination.

^d^Abnormal chest radiogram included any infiltrate, cavernous lesions, miliary pattern, or pleural effusion.

### Sensitivity, Specificity, and Predictive Values of Clinical, Radiographic, and Sonographic Signs

Table 4 shows sensitivity and specificity for the most important clinical and FASH signs. The combination ≥1 original FASH sign, abnormal chest x-ray, and body temperature ≥37.5°C had a sensitivity of 99.1% (95% CI, 94.9–99.9), a specificity of 35.2% (95% CI, 22.7–49.4), a positive predictive value of 75.2% (95% CI, 71.3–78.7), and a negative predictive value of 95.0% (95% CI, 72.3–99.3). This did not change substantially if the composite outcome confirmed or probable tuberculosis versus no tuberculosis was analyzed (see Supplementary Table S6).

**Table 4.** Sensitivity, Specificity, Predictive Values, and Accuracy of the Most Important Test Combinations Predicting Confirmed Tuberculosis (n = 166)

Predictor	Sensitivity	Specificity	PPV	NPV	Accuracy
	% (95% CI)	% (95% CI)	% (95%CI)	% (95%CI)	% (95% CI)
≥1 FASH sign	55.5 (45.7–64.9)	60.6 (47.8–72.4)	70.1 (62.5–76.8)	44.9 (38.5–52.1)	57.4 (49.7–64.8)
Abnormal chest x-ray	88.8 (81.2–94.1)	44.4 (30.9–58.6)	76.0 (71.2–80.2)	66.7 (52.1–78.7)	73.9 (66.4–80.5)
Measured T ≥37.5°	41.8 (32.5–51.6)	96.4 (87.7–99.6)	95.8 (85.3–98.9)	45.8 (41.7–49.9)	60.2 (52.5–67.7)
Constitutional symptoms^a^	96.3 (90.9–98.9)	17.0 (8.1–29.8)	70.5 (67.8–73.1)	69.2 (42.1–87.5)	70.4 (62.7–77.3)
Cough	94.6 (88.5–97.9)	12.5 (5.2–24.1)	68.0 (65.6–70.3)	53.9 (29.2–76.8)	66.9 (59.2–74.0)
Lymphadenopathy in clinical exam	63.6 (53.9–72.6)	57.1 (43.2–70.3)	74.5 (67.6–80.3)	44.4 (36.4–52.8)	61.4 (53.6–68.9)
Constitutional symptoms^a^ and T ≥37.5°	41.3 (31.9–51.1)	96.2 (87.0–99.5)	95.7 (85.0–98.9)	44.4 (40.3–48.5)	59.3 (51.3–66.9)
≥1 FASH sign and T ≥37.5°	75.5 (66.3–83.2)	67.9 (54.0–79.7)	82.2 (75.6–87.3)	58.5 (49.2–67.2)	72.9 (65.5–79.5)
Abnormal chest x-ray and T ≥37.5°	92.5 (85.8–86.7)	42.6 (29.3–56.8)	76.2 (71.6–80.2)	74.2 (57.9–85.7)	75.8 (68.4–82.2)
≥1 FASH sign and abnormal chest x-ray	98.1 (93.4–99.8)	37.0 (24.3–51.3)	75.5 (71.5–79.2)	90.9 (70.8–97.6)	77.6 (70.4–83.8)
≥1 FASH sign and abnormal chest x-ray and T ≥37.5°	99.1 (94.9–99.9)	35.2 (22.7–49.4)	75.2 (71.3–78.7)	95.0 (72.3–99.3)	77.6 (70.4–83.8)

Abbreviations: CI, confidence interval; FASH, focused assessment with sonography for HIV-associated tuberculosis; NPV, negative predictive value; PPV, positive predictive value; T, body temperature.

NOTE: ≥1 FASH sign, presence of at least 1 original FASH sign.

^a^History of weight loss, night sweat, or fever.

### Microbiological Results in Patients With Confirmed Tuberculosis

Of 110 patients with a microbiologically confirmed tuberculosis, 80 were sputum-positive. Thirty patients had no sputum or a negative sputum assay. Of these 30 patients, 5 had a positive Xpert MTB/RIF assay in urine, 3 had a positive assay in pleural fluid, and 3 had a positive assay in lymph node aspirates. Positive sputum cultures were found in 9 patients with an initially negative Xpert MTB/RIF assay, confirmed to be *M tuberculosis*. In 7 patients, tuberculosis was diagnosed by elevated ADA alone (3 in pleural fluid, 2 in pericardial fluid, and 2 in ascitic fluid). One patient tested positive only on pleural fluid culture, and 1 patient tested positive only on ascites culture. One patient had only a positive acid-fast bacilli microscopy (see Supplementary Table S7). We did not note any adverse events after puncturing procedures.

## DISCUSSION

In this prospective, observational cohort study of HIV-positive and -negative patients with suspected tuberculosis, we found that the combination of ≥1 original FASH sign, an abnormal chest-x ray, and a body temperature of ≥37.5°C had an excellent sensitivity and negative predictive value for tuberculosis. All but 1 patient with hypoechogenic splenic or hepatic lesions had confirmed tuberculosis. The 2 most common FASH signs, abdominal lymphadenopathy and pleural effusion, were associated with tuberculosis, the latter with the composite outcome confirmed or probable tuberculosis only. This composite outcome is clinically relevant, because culture as the gold standard is not always available, and its sensitivity is suboptimal [22, 23].

The positive association between hypoechogenic splenic or hepatic lesions, abdominal lymphadenopathy, and pleural effusion with tuberculosis is in agreement with a recently published study from India where 27% of 285 patients with confirmed or probable tuberculosis had pleural effusion, 14% had abdominal lymphadenopathy, and 6% had splenic or liver lesions [15]. The major difference to our study was the comparably low proportion of HIV-infected patients (20%) and the lack of follow-up by ultrasound in that study. Previous studies in HIV-positive patients in South Africa and Italy have reported even higher rates of FASH signs with 76% abdominal lymphadenopathy and 41% spleen and 31% liver lesions [12, 13]. However, these populations were small, and microbiologically confirmed tuberculosis was present in only a minority of patients.

Although pericardial effusion was more than twice as frequent in patients with tuberculosis as in patients without tuberculosis, we could not demonstrate a significant association in our study. Tuberculosis has been reported as the cause of pericardial effusions in 70% of South African patients, indicating other possible causes, eg, viral, malignancy, or connective tissue disease [24]. Thickened ileum wall might have been caused by other bacterial infections or chronic inflammatory diseases, and it was not associated with tuberculosis in our study. Ascites has a broad differential diagnosis, eg, pelvic inflammatory disease, liver or kidney disease, heart failure, or cancer [25, 26]. Thus, other diseases than tuberculosis should be considered and looked for in a patient with thickened ileum wall or ascites.

Fever and upper lobe infiltrate in chest x-ray are known signs of tuberculosis and were the strongest predictors of pulmonary tuberculosis in hospitalized HIV-positive and -negative patients in North America [27, 28]. Besides presence of cough and night sweats, fever of any duration was a predictor for tuberculosis in HIV-positive outpatients in Southeast Asia [29].

In contrast to the high sensitivity of the combination of FASH, chest x-ray, and body temperature, specificity of this combination and of most FASH signs was poor. Although the absence of sonographic and radiographic signs combined with a temperature <37.5°C might lower the probability of tuberculosis substantially, the presence of those signs would not confirm it. On the other hand, Xpert MTB/RIF assay has a high specificity of up to 99% in sputum and body fluids, and it is easy to handle even under difficult working environments [30]. However, the sensitivity of the Xpert MTB/RIF assay is reduced in HIV-positive individuals, patients with paucibacillary disease, and in extrapulmonary tuberculosis with low amounts of mycobacteria in pleural and other body fluids [4–7]. In these circumstances, overtreatment would not be reduced by implementing the Xpert MTB/RIF assay only, especially in an environment of high pretest probability for tuberculosis [2]. Thus, a diagnostic process including the combination of clinical, sonographic, radiographic, and microbiological findings might increase the rate of appropriate decisions on whether to treat patients with antituberculosis drugs.

In case of negative microbiology, normal chest x-ray, and absent FASH signs or presence of ascites only, empirical antituberculosis therapy might be safely withheld, and further tests such as full abdominal sonography and laboratory tests to examine for other diseases than tuberculosis might be done. In presence of pleural effusion, abdominal lymphadenopathy, or pericardial effusion, empirical antituberculosis therapy might be considered and started if tuberculoma in liver or spleen were detected. This is being studied in a randomized controlled trial (registration number: PACTR201712002829221).

Our study had limitations. First, 70 patients were excluded because of insufficient information or loss to follow-up. The 64 patients lost to follow-up resembled the group of patients with tuberculosis. Therefore, independent predictors for tuberculosis remained stable in the statistical models. Second, because the study was observational, the performance of FASH might have affected physicians’ treatment choices, because the results were not blinded. Third, we could not verify tuberculosis as the cause for every single FASH sign in a patient with confirmed disease, because confirmed tuberculosis was defined as ≥1 positive microbiological result, regardless where the sample was taken from. Furthermore, we cannot exclude (1) other disease agents in patients with probable tuberculosis or (2) drug resistance in patients who were classified as having no tuberculosis and did not improve on antituberculosis therapy. However, we did not find any drug resistance in our study population, and the estimated percentage of multidrug resistance cases in Tanzania is only 0.9% among new tuberculosis cases [1]. Fourth, we were not able to perform an analysis stratified by pulmonary and extrapulmonary tuberculosis, because three fourths of the patients had both pulmonary and extrapulmonary tuberculosis. Fifth, the study was done in a referral center, where the patient population might differ from primary care centers. Thus, results should be interpreted in this context. Finally, this was a single-center study done in rural Tanzania, and our results might not be generalizable to centers in higher-income countries.

## CONCLUSIONS

In conclusion, the combination of ≥1 original FASH sign, an abnormal chest x-ray, and a body temperature of ≥37.5°C had an excellent sensitivity and negative predictive value for tuberculosis. Its absence may substantially decrease the probability of tuberculosis in HIV-positive and -negative patients with clinically suspected pulmonary or extrapulmonary tuberculosis. This may help clinicians in deciding whether to withhold empirical antituberculosis therapy and might reduce overtreatment of tuberculosis.

## Supplementary Data

Supplementary materials are available at *Open Forum Infectious Diseases* online. Consisting of data provided by the authors to benefit the reader, the posted materials are not copyedited and are the sole responsibility of the authors, so questions or comments should be addressed to the corresponding author.

ofz154_suppl_Supplementary_Information
