# Supplementary material for: Sonography to Rule Out Tuberculosis in Sub-Saharan Africa: A Prospective Observational Study
Source: Open Forum Infect Dis. 2019 Apr 25;6(4):ofz154. doi: 10.1093/ofid/ofz154 (PMC6483805; doi:10.1093/ofid/ofz154)
Supplement: ofz154_suppl_Supplementary_Information [file ofz154_suppl_supplementary_information.docx]

**Supplementary Tables**

**Table S1: Description of the FASH Protocol**

| The patient is placed in supine position. Sonographic examination is done with a convex abdominal probe, except the ileum, which is done with a linear probe. FASH is performed as follows: | |
| --- | --- |
| 1. | Examine the spleen and look for hypoechogenic lesions |
| 2. | Look for free fluid in the splenorenal pouch |
| 3. | Shift the probe cranially and look for free fluid in the left pleural space |
| 4. | Put the probe in transverse position and look for pericardial effusion |
| 5. | Examine the liver and look for hypoechogenic lesions |
| 6. | Examine the retroperitoneal space for enlarged paraaortic lymphnodes >1.5cm, and look for mesenteric lymph nodes. |
| 7. | Shift the probe to the Morrison pouch and look for free fluid |
| 8. | Shift the probe cranially and look for free fluid in the left pleural space |
| 9. | Shift the probe to the pelvic region and look for free fluid in the pouch of douglas |
| 10. | Change probe to linear probe and examine the ileum |
| 11. | Let the patient sit up and look again for pleural fluid in sitting position |

Adapted from Heller et al, Focused assessment with sonography for

HIV-associated tuberculosis (FASH): a short protocol and a pictorial review. Critical Ultrasound Journal 2012, 4:21 <http://www.criticalultrasoundjournal.com/content/4/1/21>

**Table S2. Patient’s baseline characteristics: Composite outcome confirmed or probable Tuberculosis versus no Tuberculosis**

| **Variable** | | **Confirmed/probable tuberculosis**  N=135 | | **No tuberculosis**  N=56 | | **p-value** |
| --- | --- | --- | --- | --- | --- | --- |
|  | | **N** | **% or IQR** | **N** | **% or IQR** |  |
| Median age, years | | 36.5 | 27.1-44. | 41.5 | 34.2-57.3 | **0.002** |
| Male sex | | 82 | 60.7 | 25 | 44.6 | **0.041** |
| Body Mass Index, Kg/m2 | | 19.0 | 16.9-20.9 | 21.2 | 19.1-23.8 | **<0.001** |
| HIV infection | | 64 | 47.4 | 36 | 64.3 | **0.033** |
| Symptoms | Fever | 94 | 69.6 | 36 | 64.3 | 0.471 |
|  | Cough | 127 | 94.1 | 49 | 87.5 | 0.124 |
|  | Haemoptysis | 24 | 17.8 | 10 | 17.9 | 0.990 |
|  | Dyspnoea | 72 | 53.3 | 30 | 53.6 | 0.976 |
|  | Chest pain | 85 | 62.9 | 40 | 71.4 | 0.263 |
|  | Night sweats | 89 | 65.9 | 29 | 51.8 | 0.067 |
|  | Weight loss | 106 | 79.7 | 33 | 62.3 | **0.014** |
|  | Abdominal symptoms | 56 | 41.5 | 40 | 71.4 | **<0.001** |
|  | Neurological symptoms | 33 | 24.4 | 15 | 26.8 | 0.734 |
| Signs | Median temperature, °C | 37.3 | 36.4-38.0 | 36.6 | 36.0-37.0 | **<0.001** |
|  | Median SaO2,% | 97 | 95-98 | 98 | 96-98 | **0.039** |
|  | Pulmonary signs^*^ | 96 | 71.1 | 28 | 50.0 | **0.005** |
|  | Cardiac signs^**^ | 12 | 8.9 | 6 | 10.7 | 0.694 |
|  | Abdominal signs^***^ | 78 | 57.8 | 33 | 58.9 | 0.883 |
|  | Lymphadenopathy^****^ | 85 | 62.9 | 24 | 42.9 | **0.011** |
| Chest  X-ray | Upper lobe infiltrates | 74 | 54.8 | 12 | 21.4 | **<0.001** |
|  | Cavernous lesion | 38 | 28.2 | 1 | 1.8 | **<0.001** |
|  | Miliary infiltrates | 3 | 2.2 | 1 | 1.8 | 0.664 |
|  | Other infiltrates | 74 | 54.8 | 21 | 37.5 | **0.077** |
|  | Pleural effusion | 39 | 29.3 | 6 | 10.7 | **0.021** |
| Clinical tuberculosis | PTB | 26 | 19.3 | 15 | 26.8 | **0.015** |
|  | EPTB | 2 | 1.5 | 5 | 8.9 |  |
|  | PTB and EPTB | 107 | 79.3 | 36 | 64.3 |  |
| Original  FASH  signs | Pleural effusion | 41 | 30.4 | 6 | 10.7 | **0.004** |
|  | Pericardial effusion | 26 | 19.3 | 5 | 8.9 | 0.057 |
|  | Ascites | 29 | 21.5 | 11 | 19.6 | 0.776 |
|  | Abdominal LN | 27 | 20.0 | 2 | 3.6 | **0.002** |
|  | Tuberculoma (liver/spleen) | 11 | 8.2 | 0 | - | - |
|  | Ileum wall thickening | 7 | 5.2 | 3 | 5.4 | 0.604 |
|  | Ileum wall destruction | 7 | 5.2 | 4 | 7.1 | 0.410 |
| Additional sonographic signs | Splenomegaly | 34 | 25.2 | 16 | 29.1 | 0.579 |
|  | Hepatomegaly | 72 | 53.3 | 20 | 35.7 | **0.027** |
|  | Pleural fibrin | 24 | 17.8 | 2 | 3.6 | **0.005** |
|  | Pericardial fibrin | 3 | 2.2 | 0 | - | - |
| ≥1 original FASH sign | | 82 | 60.7 | 16 | 28.6 | **<0.001** |
| ≥ 1 sonographic sign (all) | | 112 | 82.9 | 38 | 67.9 | 0.061 |
| Number of sonographic signs | 0 | 23 | 17.0 | 18 | 32.1 | **0.038** |
|  | 1 | 44 | 32.6 | 21 | 37.5 |  |
|  | 2 | 22 | 16.3 | 7 | 12.5 |  |
|  | ≥3 | 46 | 34.1 | 10 | 17.9 |  |

PTB pulmonary tuberculosis; EPTB extrapulmonary tuberculosis; FASH Focused Assessment with Sonography for HIV associated Tuberculosis; SaO2 oxygen saturation. ^*^Pulmonary signs included crackles, wheezing, pleural friction in lung auscultation, or dullness in lung percussion. ^**^Cardiac signs were dilated jugular veins, lateralised apex beat, or heart murmur. ^***^Abdominal signs included organomegaly, ascites, abnormal bowel sound. ^****^Lymphadenopathy was diagnosed if palpable enlarged axillary, cervical, or nuchal lymph nodes were present on physical examination.

**Table S3: Baseline characteristics of the study population including lost to follow-up according to diagnosis of tuberculosis.**

| **Variable** | | **Confirmed/Probable Tuberculosis** | | **No Tuberculosis** | | **Missing follow-up*** | | **p-value** |
| --- | --- | --- | --- | --- | --- | --- | --- | --- |
|  | | N=135 | | N=56 | | N=64 | |  |
|  | | N | % or IQR | n | % or IQR | n | % or IQR |  |
| Median age, years | | 36.5 | 27.1-44. | 41.5 | 34.2-57.3 | 44.3 | 35.3-59.3 | **<0.001** |
| Male sex | | 82 | 60.7 | 25 | 44.6 | 41 | 64.1 | **0.064** |
| Body Mass Index, Kg/m2 | | 19.0 | 16.9-20.9 | 21.2 | 19.1-23.8 | 19.6 | 18.2-22.7 | **<0.001** |
| HIV infection | | 64 | 47.4 | 36 | 64.3 | 32 | 50.0 | 0.099 |
| Symptoms | Fever | 94 | 69.6 | 36 | 64.3 | 44 | 68.8 | 0.766 |
|  | Cough | 127 | 94.1 | 49 | 87.5 | 60 | 93.8 | 0.265 |
|  | Haemoptysis | 24 | 17.8 | 10 | 17.9 | 11 | 17.5 | 0.998 |
|  | Dyspnoea | 72 | 53.3 | 30 | 53.6 | 31 | 48.4 | 0.789 |
|  | Chest pain | 85 | 62.9 | 40 | 71.4 | 35 | 54.7 | 0.166 |
|  | Night sweats | 89 | 65.9 | 29 | 51.8 | 41 | 64.1 | 0.176 |
|  | Weight loss | 106 | 79.7 | 33 | 62.3 | 46 | 73.0 | **0.047** |
|  | Abdominal symptoms | 56 | 41.5 | 40 | 71.4 | 34 | 53.1 | **0.001** |
|  | Neuro symptoms | 33 | 24.4 | 15 | 26.8 | 17 | 26.6 | 0.920 |
| Signs | Median temperature | 37.3 | 36.4-38.0 | 36.6 | 36.0-37.0 | 36.9 | 36.3-37.3 | **<0.001** |
|  | Median SaO2, % | 97 | 95-98 | 98 | 96-98 | 97 | 94-98 | 0.063 |
|  | Pulmonary signs | 96 | 71.1 | 28 | 50.0 | 38 | 59.4 | **0.016** |
|  | Cardiac signs | 12 | 8.9 | 6 | 10.7 | 5 | 7.8 | 0.855 |
|  | Abdominal signs | 78 | 57.8 | 33 | 58.9 | 38 | 59.4 | 0.974 |
|  | Lymphadenopathy | 85 | 62.9 | 24 | 42.9 | 34 | 53.1 | **0.033** |
| Chest x-ray | Upper lobe infiltrate | 74 | 54.8 | 12 | 21.4 | 21 | 32.8 | **<0.001** |
|  | Cavernous lesion | 38 | 28.2 | 1 | 1.8 | 5 | 7.8 | **<0.001** |
|  | Miliary infiltrates | 3 | 2.2 | 1 | 1.8 | 2 | 3.1 | 0.868 |
|  | Other infiltrates | 74 | 54.8 | 21 | 37.5 | 34 | 53.1 | 0.099 |
|  | Pleural effusion | 39 | 29.3 | 6 | 10.7 | 15 | 23.4 | **0.031** |
| Clinical TB | PTB | 26 | 19.3 | 15 | 26.8 | 14 | 21.9 | **0.093** |
|  | EPTB | 2 | 1.5 | 5 | 8.9 | 3 | 4.7 |  |
|  | PTB/EPTB mixed | 107 | 79.3 | 36 | 64.3 | 47 | 73.4 |  |
| Original  FASH  signs | Pleural effusion | 41 | 30.4 | 6 | 10.7 | 15 | 23.4 | **0.015** |
|  | Pericardial effusion | 26 | 19.3 | 5 | 8.9 | 6 | 9.4 | 0.090 |
|  | Ascites | 29 | 21.5 | 11 | 19.6 | 24 | 37.5 | **0.029** |
|  | Abdominal LN | 27 | 20.0 | 2 | 3.6 | 5 | 7.8 | **0.003** |
|  | Hypoechogenic lesions spleen/liver | 11 | 8.2 | 0 | - | 2 | 3.2 | - |
|  | Ileum wall thickening | 7 | 5.2 | 3 | 5.4 | 3 | 4.7 | 0.984 |
|  | Ileum wall destruction | 7 | 5.2 | 4 | 7.1 | 8 | 12.5 | 0.193 |
| Additional sonographic signs | Splenomegaly | 34 | 25.2 | 16 | 29.1 | 22 | 34.9 | 0.365 |
|  | Hepatomegaly | 72 | 53.3 | 20 | 35.7 | 25 | 39.1 | **0.038** |
|  | Pleural fibrin | 24 | 17.8 | 2 | 3.6 | 9 | 14.1 | **0.021** |
|  | Pericardial fibrin | 3 | 2.2 | 0 | - | 0 | - | - |
| ≥1 original FASH sign | | 82 | 60.7 | 16 | 28.6 | 37 | 57.8 | **<0.001** |
| ≥1 sonographic sign (all) | | 112 | 82.9 | 38 | 67.9 | 50 | 78.1 | 0.069 |
| number of sonographic signs | 0 | 23 | 17.0 | 18 | 32.1 | 27 | 42.2 | **0.002** |
|  | 1 | 44 | 32.6 | 21 | 37.5 | 25 | 39.1 |  |
|  | 2 | 22 | 16.3 | 7 | 12.5 | 9 | 14.1 |  |
|  | ≥3 | 46 | 34.1 | 10 | 17.9 | 3 | 4.7 |  |

TB Tuberculosis; PTB pulmonary TB; EPTB extrapulmonary TB; FASH Focused Assessment with Sonography for HIV associated Tuberculosis; Abdominal LN, abdominal lymphnodes >1.5cm. *No positive microbiology at enrolment and lost to follow-up. P-values refer to patients missed to follow up compared to patients with no tuberculosis.

**Table S4: Predictors of the composite outcome confirmed or probable tuberculosis versus no tuberculosis (n=135 vs 56, Univariate logistic regression).**

| **Variable** | | **Odds Ratios** | **95% CI** | **p-value** |
| --- | --- | --- | --- | --- |
| Age, per 10 years older | | 0.67 | 0.54-0.85 | **0.001** |
| Female versus Male | | 0.52 | 0.28-0.98 | **0.043** |
| Body Mass Index, per 5 Kg/m2 increase | | 0.37 | 0.23-0.59 | **<0.001** |
| HIV infection | | 0.50 | 0.26-0.95 | **0.035** |
| Fever | | 1.27 | 0.66-2.46 | 0.471 |
| Cough | | 2.27 | 0.78-6.58 | 0.132 |
| Haemoptysis | | 0.99 | 0.44-2.24 | 0.990 |
| Dyspnoea | | 0.99 | 0.53-1.85 | 0.976 |
| Chest pain | | 0.68 | 0.36-1.33 | 0.264 |
| Night sweats | | 1.80 | 0.96-3.39 | 0.069 |
| Weight loss | | 2.38 | 1.18-4.78 | **0.015** |
| Abdominal symptoms | | 0.28 | 0.14-0.56 | **<0.001** |
| Neurological symptoms | | 0.88 | 0.43-1.80 | 0.734 |
| Temperature, per each °C increase | | 2.55 | 1.68-3.85 | **<0.001** |
| Sao2 | | 0.89 | 0.78-1.02 | 0.101 |
| Pulmonary signs^*^ | | 2.46 | 1.29-4.68 | **0.006** |
| Cardiac signs^**^ | | 0.81 | 0.28-2.29 | 0.695 |
| Abdominal signs^***^ | | 0.95 | 0.51-1.80 | 0.883 |
| Lymphadenopathy**** | | 2.26 | 1.20-4.27 | **0.011** |
| Chest x-ray | Upper lobe infiltrates | 4.38 | 2.12-9.08 | **<0.001** |
|  | Cavernous lesion | 21.4 | 2.95-160.5 | **0.003** |
|  | Miliary infiltrates | 1.25 | 0.13-12.3 | 0.848 |
|  | Other infiltrates | 1.97 | 1.03-3.76 | **0.039** |
|  | Pleural effusion | 3.32 | 1.31-8.38 | **0.011** |
|  | Abnormal X-ray^£^ | 7.32 | 3.34-16.1 | **<0.001** |
| Original FASH signs | Pleural effusion | 3.63 | 1.44-9.14 | **0.006** |
|  | Pericardial effusion | 2.43 | 0.88-6.70 | 0.086 |
|  | Ascites | 1.12 | 0.51-2.43 | 0.776 |
|  | Abdominal LN | 6.75 | 1.54-29.4 | **0.011** |
|  | Mesenterial LN | 2.16 | 1.57-2.95 | **<0.001** |
|  | Ileum thick wall | 0.97 | 0.24-3.87 | 0.961 |
|  | Ileum thick wall dx | 0.71 | 0.20-2.53 | 0.599 |
| Additional sonographic signs | Splenomegaly | 0.82 | 0.41-1.65 | 0.580 |
|  | Hepatomegaly | 2.06 | 1.08-3.91 | **0.010** |
|  | Pleural fibrin | 5.84 | 1.33-25.6 | **0.019** |
|  | Pericardial fibrin | - | - | - |
| ≥1 original FASH sign^$^ | | 3.87 | 1.96-7.59 | **<0.001** |
| Number of sonographic signs^$$^ | 0 | - | - | - |
|  | 1 | 1.64 | 0.73-3.67 | 0.230 |
|  | 2 | 2.46 | 0.86-7.03 | 0.093 |
|  | ≥3 | 3.60 | 1.43-9.04 | **0.006** |

PTB pulmonary tuberculosis; EPTB extrapulmonary tuberculosis; FASH Focused Assessment with Sonography for HIV associated Tuberculosis. SaO2 oxygen saturation; LN lymphadenopathy

^*^ Pulmonary signs included crackles, wheezing, pleural friction in lung auscultation, or dullness in lung percussion ; ^**^ Cardiac signs included dilated jugular veins, lateralised apex beat, or heart murmur; ^***^ Abdominal signs were organomegaly, ascites, abnormal bowel sounds; ^****^ Lymphadenopathy included palpable enlarged axillary, cervical, or nuchal lymph nodes on physical examination. ^£^ abnormal chest x-ray included any of above radiological signs, ^$^ Presence of at least one of the above mentioned original FASH signs. ^$$^ Presence of at least one original FASH criteria and/or splenomegaly, hepatomegaly, or pleural- or pericardial fibrin strands

**Table S5. Predictors of the composite outcome confirmed or probable tuberculosis (n=135 vs 56). Multivariate logistic regression.**

**Adjusted for all variables listed*

| **Variable** | **Odds Ratios** | **95% CI** | **p-value** |
| --- | --- | --- | --- |
| Age, per 10 years older | 0.70 | 0.51-0.97 | **0.030** |
| Female versus Male | 1.01 | 0.39-2.67 | 0.971 |
| Body Mass Index, per 5 Kg/m2 increase | 0.45 | 0.21-1.07 | 0.072 |
| HIV infection | 0.66 | 0.23-1.89 | 0.443 |
| Cough | 2.55 | 0.79-7.76 | 0.120 |
| Night sweats | 0.58 | 0.25-1.84 | 0.452 |
| Weight loss | 0.76 | 0.22-1.54 | 0.278 |
| Temperature, per each °C increase | 2.48 | 1.43-4.30 | **0.001** |
| Pulmonary signs^*^ | 1.09 | 0.39-3.05 | 0.859 |
| Abdominal signs^**^ | 0.71 | 0.26-1.90 | 0.493 |
| Lymphadenopathy^***^ | 1.95 | 0.70-5.41 | 0.198 |
| Abnormal chest x-ray^****^ | 8.45 | 2.68-27.6 | **<0.001** |
| ≥1 original FASH sign | 4.62 | 1.69-12.6 | **0.003** |

Odds ratios were adjusted for all variables listed. CI confidence interval ;

^*^Pulmonary signs included crackles, wheezing, pleural friction in lung auscultation, or dullness in lung percussion. ^**^Abdominal signs included organomegaly, ascites, abnormal bowel sound. ^***^Lymphadenopathy was diagnosed if palpable enlarged axillary, cervical, or nuchal lymph nodes were present on physical examination. ^****^Abnormal chest x-ray included any infiltrate, cavernous lesions, miliary pattern or pleural effusion

**Table S6: Sensitivity, Specificity, Predictive Values, and Accuracy of the most important test combinations predicting confirmed or probable tuberculosis (n=191)**

|  | **Sensitivity**  **% (95% CI)** | **Specificity**  **% (95% CI)** | **PPV**  **% (95% CI)** | **NPV**  **% (95% CI)** | **Accuracy**  **% (95%CI)** |
| --- | --- | --- | --- | --- | --- |
| ≥1 FASH sign | 60.7 (51.9-69.0) | 71.4 (57.8-82.7) | 83.7 (76.8-88.8) | 43.0 (36.6-49.7) | 63.9 (56.6-70.7) |
| Abnormal chest x-ray | 90.2 (83.8-94.7) | 44.4 (30.9-58.6) | 79.9 (75.6-83.5) | 64.9 (50.4-77.0) | 76.9 (70.2-82.7) |
| Measured T ≥37.5°C | 39.3 (31.0-48.0) | 96.4 (87.7-99.6) | 96.4 (86.9-99.1) | 39.7 (36.3-43.2) | 56.0 (48.7-63.2) |
| Constitutional symptoms^*^ | 96.9 (92.5-99.2) | 17.0 (8.1-29.8) | 74.6 (72.1-76.9) | 69.2 (42.0-87.5) | 74.2 (67.3-80.3) |
| Cough | 94.1 (88.7-97.4) | 12.5 (5.2-24.1) | 72.2 (70.0-74.3) | 46.7 (25.0-69.7) | 70.2 (63.1-76.6) |
| Lymphadenopathy in clinical exam | 62.9 (54.2-71.1) | 57.1 (43.2-70.3) | 78.0 (71.8-83.1) | 39.0 (31.8-46.8) | 61.3 (53.9-68.2) |
| ≥1 FASH sign and T ≥37.5° | 77.8 (69.8-84.5) | 67.9 (54.0-79.7) | 85.4 (79.8-89.6) | 55.9 (46.8-64.6) | 74.9 (68.1-80.9) |
| Abnormal chest x-ray and T ≥37.5° | 93.9 (88.4-97.4) | 42.6 (29.2-56.8) | 80.0 (76.0-83.5) | 74.2 (57.9-85.8) | 79.0 (72.5-84.6) |
| ≥1 FASH sign and abnormal x-ray | 98.5 (94.6-99.8) | 37.0 (24.3-51.3) | 79.3 (75.7-82.5) | 90.9 (70.8-97.6) | 80.7 (74.2-86.1) |
| ≥1 FASH sign and abnormal chest x-ray and T ≥37.5° | 99.2 (95.9-99.9) | 35.2 (22.7-49.4) | 78.9 (74.5-82.0) | 95.0 (72.3-99.3) | 80.7 (74.2-86.1) |

PPV, positive predictive value; NPV, negative predictive value; CI, confidence interval; FASH, Focused Assessment with Sonography for HIV associated Tuberculosis; T, body temperature; * history of weight loss, night sweat or fever; ≥1 FASH sign: presence of any original FASH signs.

**Table S7: Microbiological results in 110 patients with confirmed tuberculosis**

|  | **PTB**  **N = 24** | **EPTB**  **N = 2** | **EPTB/PTB**  **N = 84** | **All patients**  **N = 110** |
| --- | --- | --- | --- | --- |
| **Sputum samples positive tests**  *(positive tests/tests done)*   - Xpert positive - Culture positive only - AFB positive | N  22/24  2/24  3/3 | N  0/1  0/0  0/0 | N  58/72  5/75  9/9 | N  80/97  7/99  12/12 |
| **EPTB samples positive tests**  *(positive tests/tests done)*   - Xpert Urine - Xpert Pleural effusion - Xpert Pericardial effusion - Xpert Lymph node aspiration - Xpert ascites - ADA pos. pleural fluid - ADA pos. ascites - ADA pos. pericardial fluid - Culture pos. pleural fluid - Culture pos. ascites - Culture pos. pericardial fluid - Culture pos. lymph node aspirate | 0/21  0/0  0/0  0/0  0/0  0/0  0/0  0/0  0/0  0/0  0/0  0/0 | 0/1  0/0  0/0  0/0  0/2  0/0  2/2  0/0  0/0  0/0  0/0  0/0 | 14/79  3/17  0/4  4/4  1/8  3/6  1/5  3/3  4/14  1/5  0/3  2/2 | 14/101  3/17  0/4  4/4  1/10  3/6  3/7  3/3  4/14  1/5  0/3  2/2 |

PTB, pulmonary tuberculosis; EPTB, extrapulmonary tuberculosis; EPTB/PTB, extrapulmonary and pulmonary tuberculosis. AFB, acid-fast bacilli. ADA pos, adenosine deaminase (ADA) ≥40 U/ml in pleural fluid (20), ≥35 U/ml in pericardial fluid (4) and ≥30 U/ml in ascitic fluid (21); Culture pos, growth of *Mycobacterium tuberculosis* in culture.
